# A Flexible Ultrasound Transducer Array with Micro-Machined Bulk PZT

**DOI:** 10.3390/s150202538

**Published:** 2015-01-23

**Authors:** Zhe Wang, Qing-Tang Xue, Yuan-Quan Chen, Yi Shu, He Tian, Yi Yang, Dan Xie, Jian-Wen Luo, Tian-Ling Ren

**Affiliations:** 1 Institute of Microelectronics, Tsinghua University, Beijing 100084, China; E-Mails: wangzhe.thu2012@gmail.com (Z.W.); qingtang_xue@163.com (Q.-T.X.); chenyq.thu@gmail.com (Y.-Q.C.); shuyithuphy@gmail.com (Y.S.); tianh10@mails.tsinghua.edu.cn (H.T.); yiyang@tsinghua.edu.cn (Y.Y.); xiedan@tsinghua.edu.cn (D.X.); 2 Tsinghua National Laboratory for Information Science and Technology (TNList), Beijing 100084, China; 3 Department of Biomedical Engineering, Tsinghua University, Beijing 100084, China; E-Mail: luo_jianwen@tsinghua.edu.cn

**Keywords:** ultrasonic, flexible ultrasound transducer, ultrasound imaging, flexible device, PZT

## Abstract

This paper proposes a novel flexible piezoelectric micro-machined ultrasound transducer, which is based on PZT and a polyimide substrate. The transducer is made on the polyimide substrate and packaged with medical polydimethylsiloxane. Instead of etching the PZT ceramic, this paper proposes a method of putting diced PZT blocks into holes on the polyimide which are pre-etched. The device works in d31 mode and the electromechanical coupling factor is 22.25%. Its flexibility, good conformal contacting with skin surfaces and proper resonant frequency make the device suitable for heart imaging. The flexible packaging ultrasound transducer also has a good waterproof performance after hundreds of ultrasonic electric tests in water. It is a promising ultrasound transducer and will be an effective supplementary ultrasound imaging method in the practical applications.

## 1. Introduction

Ultrasound transducers are core modules of medical imaging systems. Traditional ultrasound transducers are all based on rigid substrates [1–4], while two-dimension arrays do not conform to the human body shape and line-arrays are difficult to operate. At the same time, transducers cannot be used for implant ultrasound imaging [5,6]. A variety of flexible ultrasound transducers have been reported recently. Mastronardi *et al.* proposed a flexible ultrasound transducer made of AlN [7], and Zhou *et al.* mentioned that they took advantage of the properties of ZnO nanowires to make a flexible ultrasound transducer [8]. However, these materials do not have as good performance as lead zirconate titanate (PZT, Pb(Zr_1−x_Ti_x_)O_3_). Bernstein *et al.* fabricated monomorph sonar transducers using sol-gel PZT films up to 12 μm in thickness and successfully generated high-resolution images at a range of two meters underwater using 8 × 8 and 6 × 6 arrays [9]. Another piezoelectric micro-fabricated ultrasound transducer (pMUT) structure driven by a sol-gel-deposited PZT layer up to 4 μm thick was built, tested, and simulated by Muralt *et al.* [10]. However, the piezoelectricity of the sol-gel PZT film is not as good as that of PZT ceramic, so the performance of the transducer has room for improvement. Some researchers have already demonstrated initial imaging results from both one-dimensional (1D) and 2D capacitive micro-fabricated ultrasound transducers (cMUT) [11,12]. Although cMUTs have an almost perfect theoretical coupling factor [13], there are practical operational and fabrication considerations that limit the achievable coupling. The cMUT array has good conformity, impedance matching and high coupling factor, but the high working voltage limits its application in medical fields. The rigid ultrasound transducer also does not fit the body's skin very much and when used in some surgical operations nurses' help is needed to fix the transducer to the patient's chest, so the flexible transducer concept was introduced. Bernstein *et al.* [14] also fabricated 2D arrays of square and rectangular pMUT membranes driven by PZT. Dausch *et al.* [15] fabricated pMUT arrays to detect intracardiac echoes. Jiang *et al.* [16–19] proposed a PMN-PT-based ultrasound array to make the ultrasound transducer flexible, but because the PMN-PT composite is not very flexible there is still room for improvement. Powell and Hayward [20] proposed a method that involves putting a long bar by an array of piezoelectric ceramic glued onto the polymer substrate to make the device flexible, but the deficiency is that their devices are not reliable. P.I. Hsu *et al.* proposed another structure that made the ultrasound transducer flexible [21]. Anthony Gachagan *et al.* mentioned that the piezoelectric ceramic can be divided firstly and then the pitch of the ceramics refilled [22], but it is difficult to meet the requirements for the precise distance between the cells. Khuri-Yakub *et al.* proposed a flexible capacitive micro-machined transducer array (cMUT) by embedding bulk PZT in a polymer matrix [23]. Grandfest *et al.* studied flexible pMUT and made progressive improvements. They etched silicon substrates into blocks with trenches, and the trenches were refilled with polydimethylsiloxane (PDMS). However, the PDMS filled in the pitch was not robust and the device needs more steeps to make it work. Flexible piezoelectric micro-fabricated ultrasound transducers (pMUT) were reported by Singh *et al.* [24,25]. They used a similar strategy as the flexible cMUT, but mounted diced PZT ceramics onto the silicon blocks. The trenches were partly refilled with polyimide (PI), which made for flexible joints, but their flexible joints were not robust enough and could not endure more substantial bending.

## 2. Design and Simulation

**Figure 1.**
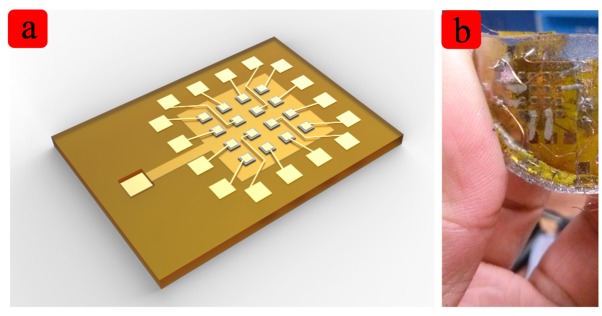
(**a**) shows the schematic diagram of the FpMUT; (**b**) The device is packaged with PDMS and it is flexible, stretchable and causes no harm to humans.

A micro-machined flexible ultrasound transducer array is reported in this paper. Prior work has been done a foreshadowing for the ultrasound transducer [26,27]. The transducer array is based on MEMS technology, so it has good uniformity and can be scaled down. The piezoelectric elements which are shown in Figure 1 are wrapped by polyimide and biomedical-grade PDMS, which is flexible, and robust. There are two reasons why we chose ∼2 MHz. Firstly, the ultrasound transducer can emitting ultrasound waves to the heart to make an image, so we can know the internal structure to identify some diseases. Secondly, ultrasound imaging is real-time imaging, so we can observe the motion of the heart, and we can detect some diseases from the abnormal movement. The traditional hard substrate ultrasound transducer for heart imaging is also about 2–3 MHz, so our flexible ultrasound transducer is also ∼2 MHz. The resonant frequency is decided by many parameters. The main parameters are the size of the PZT, the thickness of the PZT, the thickness of polyimide and PDMS and so on. The experiments show as good performance of the ultrasound transducer as that of the traditional ones. The transducer is well characterized, and has potential applications in medical imaging, especially in real-time surgery.

The materials are selected for the following reasons: polyimide (PI) was chosen for its good biocompatibility and flexibility. However, the Young modulus of PI is not close to that of the human body. The Young modulus and the acoustic impedance of the PDMS are close to that of the body, but it is not a good choice for the substrate of the device. This is because the surface of PDMS is not as smooth as PI and the coefficient of thermal expansion difference between the PDMS and electrode material is very large, so the substrate will be crushed when sputtering Au. Considering the above factors, PI was the most suitable material for the substrate.

**Figure 2.**
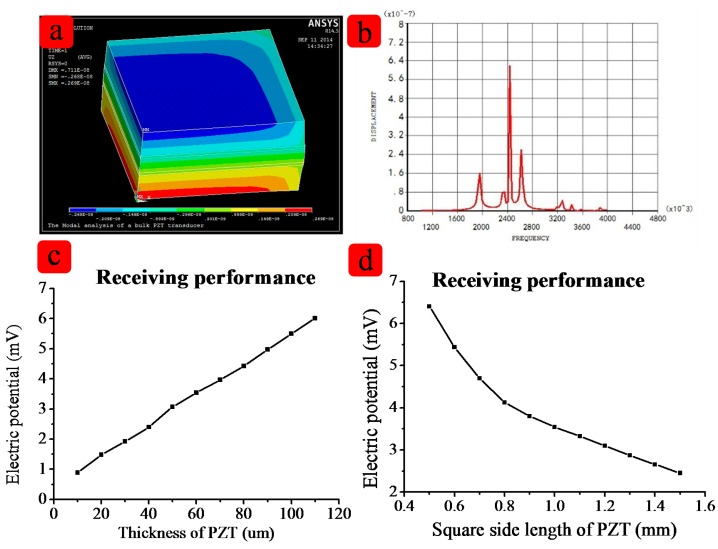
(**a**) the displacement along the Z axis. The red means the positive displacement and the blue means the negative displacement; (**b**) shows the resonant frequency of the simulation; (**c**) and (**d**) show the thickness and the size which are influence the frequency of the device.

The structure of this device was designed by the simulation tools. Table 1 lists several key features of the flexible ultrasound array. To validate the design, both Finite Element Analysis (Ansys) and direction analysis (Matlab) were carried out. Because all 16 elements are the same, so one cell was selected in the simulation, as shown in Figure 2a.

**Figure 3.**
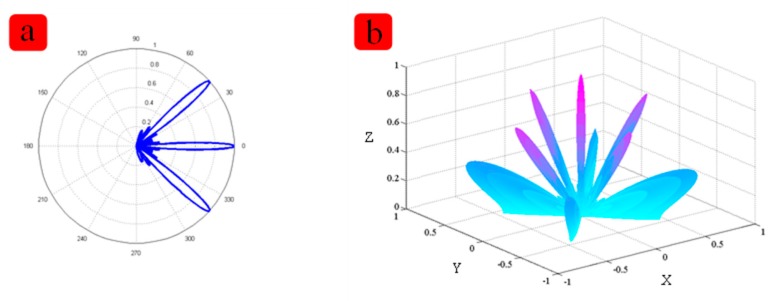
The directivity simulation results with the Matlab software. The distance between two elements in about 1.5 times the wavelength (**a**) shows the 2-dimensional figure and (**b**) shows the 3-dimensional result.

The length and width are 1 mm × 1 mm. Figure 2c shows the relationship between the thickness and the receiving performance. Figure 2d shows relationship between size and the receiving performance. In order to achieve the better performance of the device, the distance between two elements is a very important feature. Matlab modeling was applied. Figure 3 shows the simulation result about the distance between two elements. When the distance between two cells increases, the main lobe beam is becoming narrower, and the directional accuracy is becoming high, however, the side lobe will become bigger too. According to the simulation result, the effect of PDMS are very limited on the resonant frequency, but the thickness of the PDMS affects the vibration displacement, so the PDMS should not too thick. After simulation and experimenta, we chose 100 μm PDMS for our device. After the simulation, we chose 1 mm for the distance between two cells. All the parameters including the specific software module, material properties used are listed in Tables 2 and 3.

## 3. Device Fabrication

**Figure 4.**
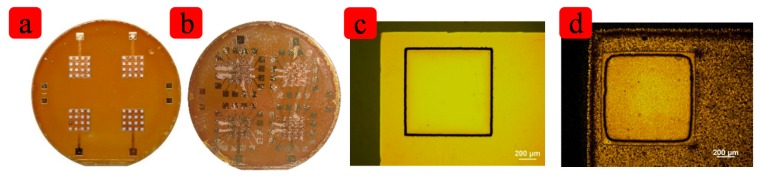
(**a**) The PZT are put into the holes of PI film; (**b**) The top electrodes are pattened; (**c**) and (**d**) show the images before and after DRIE eching.

Firstly, the polyimide thin films was spin-coated on a silicon wafer. The thickness is about 10 μm. The contact between PI film and wafer is very firm so the PI film can persist on the silicon substrate for a long time. The second step is the lithography, and then Ti/Au (200 Å/1000 Å) film was sputtered and patterned by lift-off. The third step is spin-coating of another thick PI layer and etching the holes where the PZT elements will be placed. This PI layer is about 80 μm thick. In order to pattern the PI film, another lithography process is applied and the photoresist works as a mask. Then the DRIE etching is carried out. The PI film before and after etching can be seen in the Figure 4a,b. The edge is not straight because the etching is anisotropic. In the corner, the space is limited so the chemical reactants cannot easily removed from the surface of the PI film, so the corners are not sharp any more. After 100 minutes' etching, the bottom electrode is revealed and this can be confirmed by detecting the resistance between two pads of bottom electrode. The fourth step is putting the diced PZT in the holes via silver paste. It can be seen in Figure 4c. The fifth step is to form the top electrode. The Ti/Au (200 Å/1000 Å) film was sputtered and patterned by lift-off process to form the top electrode and the pads. It can be seen in Figure 4d. The sixth step is wire-bonding. The copper wire was welded to the pad for device testing. Then the whole device is removed from the Si substrate by heating on a hotplate, with a little water on it. The PI film is hydrophobic, so it very easy to peel off from the silicon. The last step is coating the device with biomedical-grade PDMS from the top side to the bottom. This PDMS film is about 1 mm thick and is robust enough to make sure the copper wire would not break off from the device. Figure 4 lists parts process of the devices.

## 4. Device Characterization and Discussion

**Figure 5.**
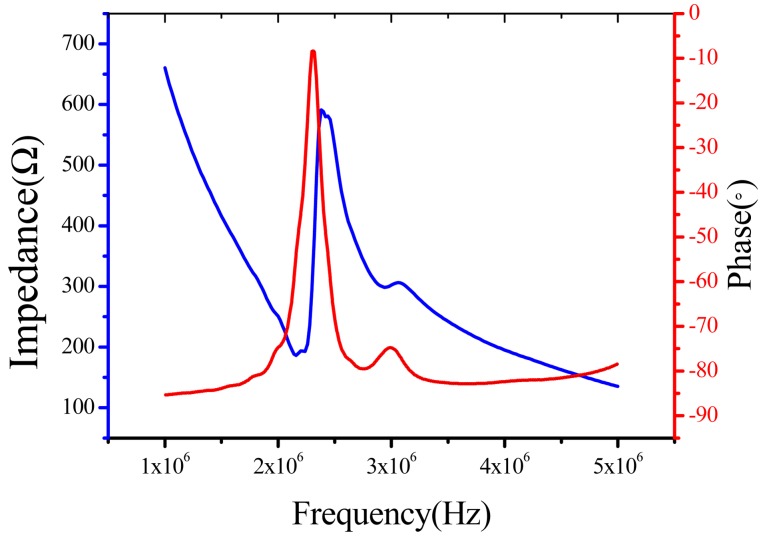
The experimental results of impedance and phase.

The device must be polarized before the test in the polydimethylsiloxane fluid at about 120 °C and 400 V DC voltage was applied for 40 min for polarization. An Agilent HP4294A precision impedance analyzer was used to measure the electrical impedance. The device was biased at a constant DC voltage and a variable AC voltage. The impedance is shown in Figure 5. The center frequency was about 2.2 MHz. The electromechanical coupling coefficient was calculated by following formula:

$$k_{\mathrm{eff}}^{2}=1-{\left(\frac{f_{s}}{f_{a}}\right)}^{2}$$

where *f_s_* is the resonant frequency, the lowest point on the characteristic impedance curve, *f_a_* is anti-resonance frequency, the highest point on the characteristic impedance curve.

The calculated electromechanical coupling coefficient factor from Figure 5 is about 0.21, which is lower than that of the bulk PZT material. This is because the device is wrapped with flexible materials like PI and PDMS so the damping effect cannot be ignored. Actually because this device is working in d31 mode, the electromechanical coupling coefficient is very considerable.

**Figure 6.**
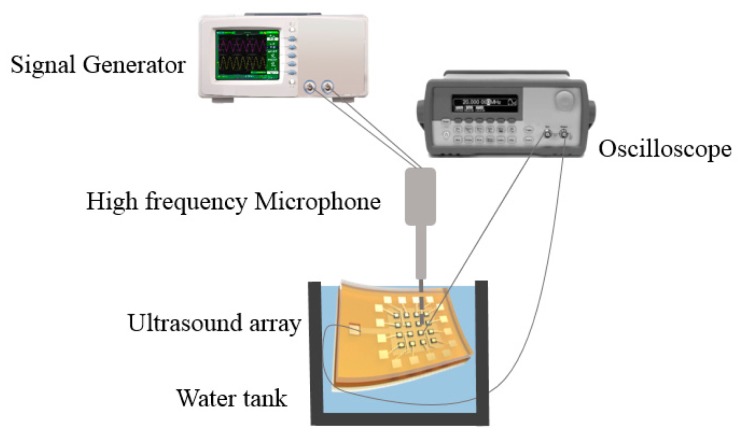
The schematic diagram of the test. The whole device tested in the water tank without any waterproof material.

The device is flexible and waterproof so the tests can be carried out in the water. The resonant frequency in water was also extracted from the measurements made using the high frequency microphone. Figure 5 shows the dependence of the open circuit resonant frequency on the DC bias voltage. The measurement results agree with the model within 5% of error. The difference can be explained by processing uncertainties, such as the gap between two cells and the PI layer and PDMS layer thickness. Another possible cause for the discrepancies is the effect of the silver paste, which is not considered in the model. The full effect of the PDMS coating on the device performance are currently under investigation both by finite element simulation and experiments. The schematic of FpMUT for ultrasonic measurement is shown in Figure 6.

**Figure 7.**
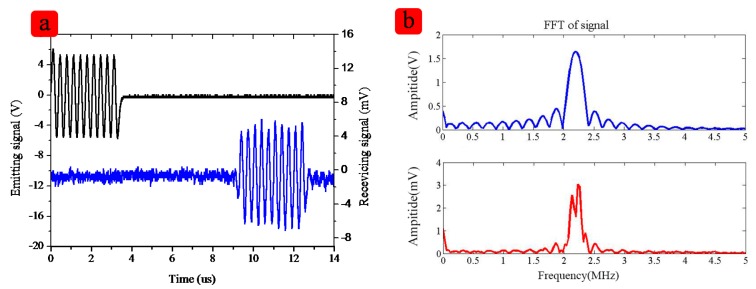
The response of one typical array element. (**a**) shows the emitting signal and the receiving signal; (**b**) shows the FFT of the signals, the spectrum signal.

The measurement system includes a signal generator, an oscilloscope and a high frequency microphone. The signal generator emits a series of signal to the flexible device and after several microseconds the high frequency microphone will receive the signal from the device. It can be seen in Figure 7b, that the Fast Fourier Transform (FFT) of the signal shows the signal received by device is the signal sent before, proving the response is caused by the emitting signal. All 16 elements show good uniformity. The emission and receiving performance for the array is summarized in Table 4.

In the water the ultrasonic velocity is about 1500 m/s, and the frequency of the device is about 2.2 MHz, the wavelength is about 0.7 mm, therefore the distance between two elements is about 1.5–2 times the wavelength, so from the result, it can be also calculated that the distance between two elements is about 1.4 cm, which is as same as the measurement. The spectrum signal of the signal has a concave shape, which shows that there are some signals missing. That is because there are some reflections between the junction of the PI film and the PDMS. However, the most valid signals can support the needs of medical imaging applications.

## 5. Conclusions

In this paper we propose a novel flexible 2-dimensional ultrasound array using bulk PZT with a resonant frequency over 2 MHz. The arrays consist of 16 elements and each element was of 1 mm × 1 mm size and with a pitch of 1 mm. The thickness is about 100 μm. The bandwidth is about 21%. The electromechanical coupling coefficient is about 22.25%. Electric impedance and pulse-echo experiments were applied. All 16 elements showed good uniformity and were robust enough to test in the water. The good flexibility and high electromechanical coupling coefficient show the high potential of the proposed device in medical applications.

## Figures and Tables

**Table 1.** Simulation parameters.

Center frequency	2 MHz
Element-to-element pitch	1 mm
Number of elements	16
Size of element	1 mm × 1 mm

**Table 2.** Finite element model of the type used in ansys.

**Structure Layer**	**Model Type**
PZT	SOLID5
Polyimide	SOLID45
PDMS	SOLID45
Electrode (Au)	SHELL41

**Table 3.** Elastic matrix parameters and piezoelectric matrix parameters of PZT.

**Stiffness Constant**	**c_11_**	**c_12_**	**c_13_**	**c_33_**	**c_44_**
(Gpa)	135	67.4	68.1	113	22.2
Piezoelectric strain constant	e_31_	e_33_	e_15_		
(C/m^2^)	−1.86	9	9.8		

**Table 4.** Measured Properties of the 16-Elment Linear Array.

Number of elements	16
Average center frequency	2.27 MHz
High/Low center frequency	2.4 MHz/2.14 MHz
